# Supplementary material for: Phosphatidylinositol 4,5-bisphosphate impacts extracellular vesicle shedding from C. elegans ciliated sensory neurons
Source: J Cell Sci. 2025 Oct 31;138(20):jcs264005. doi: 10.1242/jcs.264005 (PMC12633741; doi:10.1242/jcs.264005)
Supplement: Supplementary information [file joces-138-264005-s1.pdf]

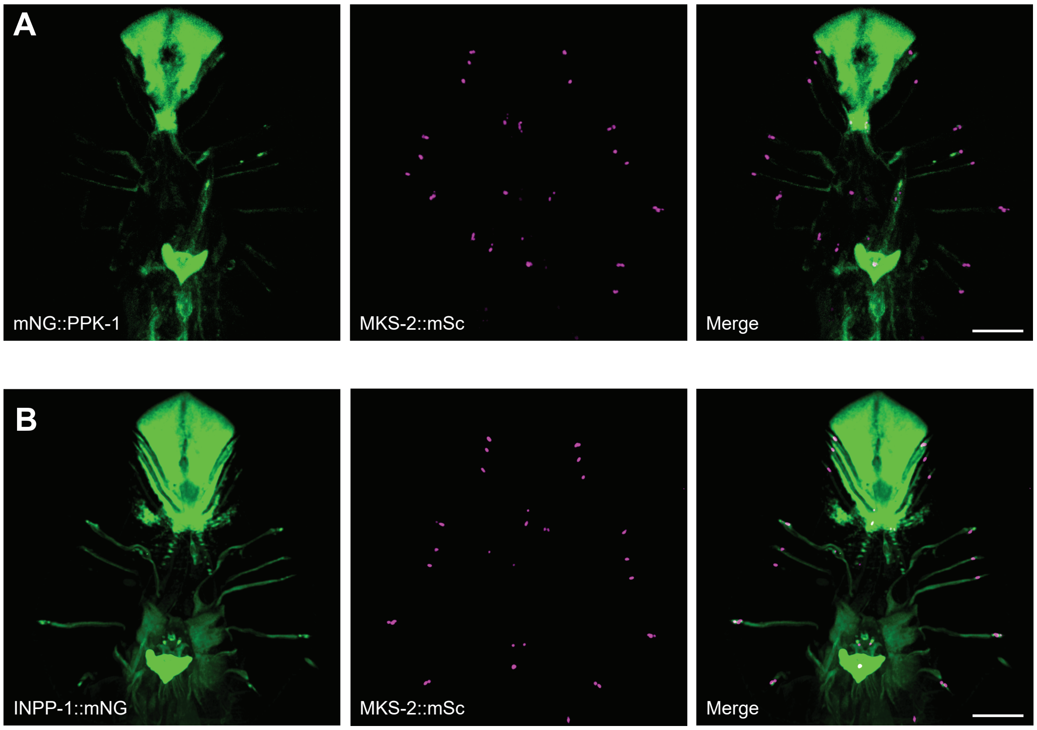

Fig. S1. Localization of PI(4,5)P$_2$ regulatory enzymes in the male tail. (A,B) Endogenously tagged (A) mNG::PPK-1 and (B) INPP-1::mNG with the MKS-2::mSc TZ marker in the RnB neurons of the male tail; scale, 10 μm.

Journal of Cell Science • Supplementary information

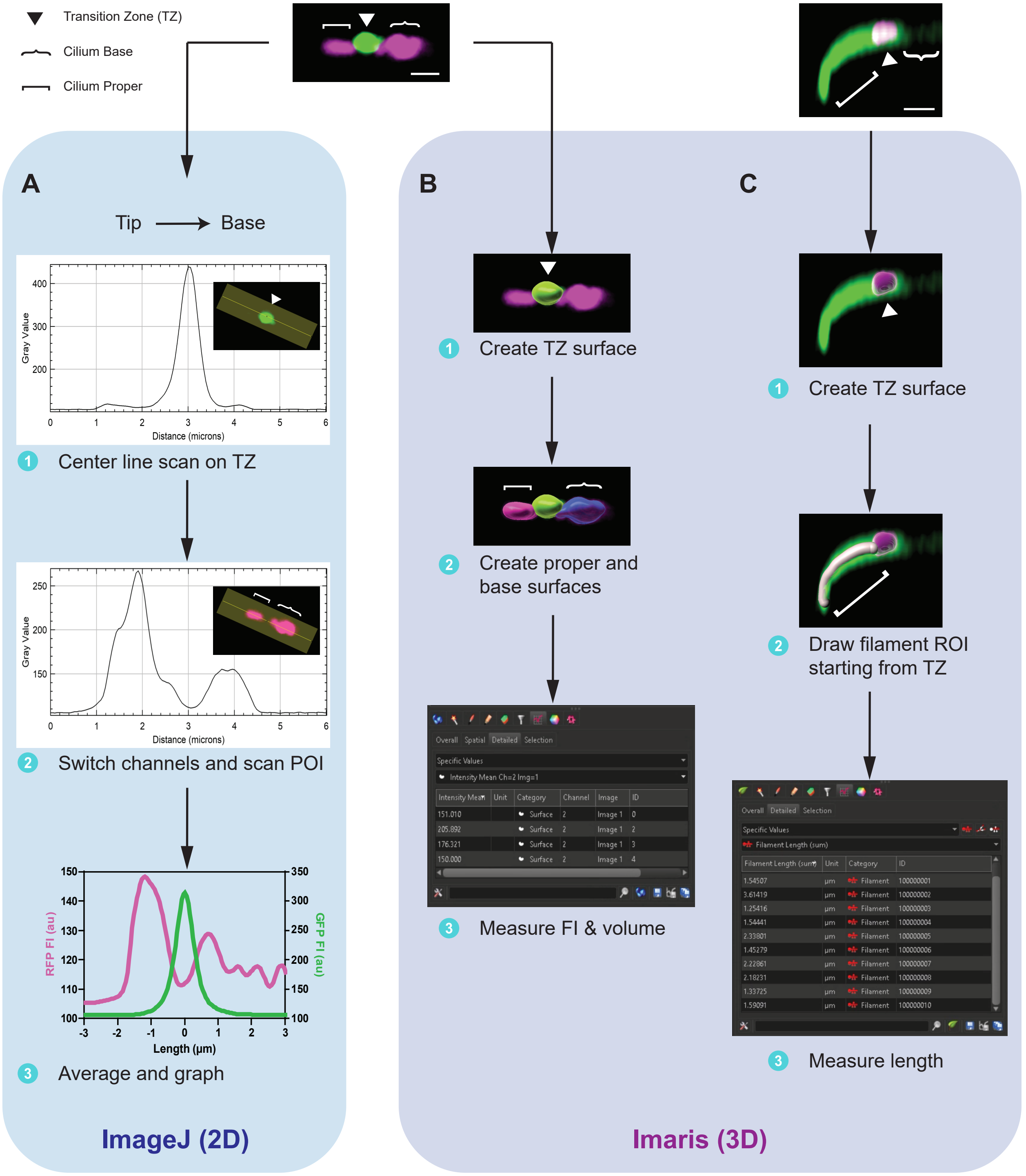

Fig. S2. Quantification of ciliary proteins. (A) Line scan analysis performed to determine distribution of mNG::PLCδ1-PH (Fig. 1B; Fig. 7F), mNG::PPK-1 (Fig. 1C), INPP- 1::mNG (Fig. 1D), PKD-2::GFP (Fig. 4E), INPP-5K::mNG (Fig. 7E) and CLHM-1::tdT (Sup. Fig. S5C) proteins of interest (POI). (B) Volumetric and fluorescence intensity analyses (Fig. 2B; Fig. 4B,C,F,H,I; Fig. 6D,E; Sup. Fig. S5A,B) and (C) measurements of cilium length (Fig. 8B,C and Sup. Fig. S7A,B) were carried out on 3D reconstructions.

Journal of Cell Science • Supplementary information

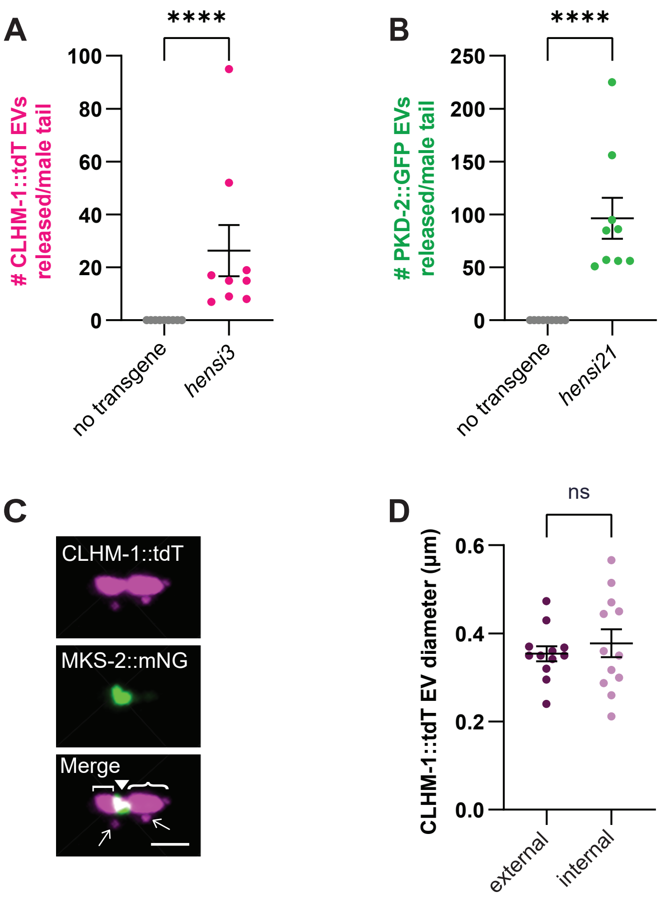

Fig. S3. CLHM-1::tdT and PKD-2::GFP label EVs released from male tail RnB neurons. (A) CLHM-1::tdTomato and (B) PKD-2::GFP labeled EVs are identified by Imaris spot detection in TIRF images of males that express the CLHM-1::tdT and PKD-2::GFP single copy transgenes, but not *him-5* control animals; n = 9. (C) Representative image of a CLHM-1::tdT-labeled ectosome (top) adjacent to the ciliary base; TZ labeled with MKS-2::mNG (middle). The ectosomes (↓), cilium proper ([), transition zone (▼), ciliary base ({), and scale, 2 μm are labeled in the merge image (bottom). (D) Quantification of the diameter of CLHM-1::tdT EVs inside the worm, adjacent to the ciliary base as in (C) versus the CLHM-1::tdT EVs released into the external environment; n = 12. No difference in size was detected, indicating that the CLHM-1::tdT EVs that bud from the ciliary base are indeed released into the external environment. Data are represented as mean +/- SEM; Mann-Whitney test, **** $p < 0.0001$.

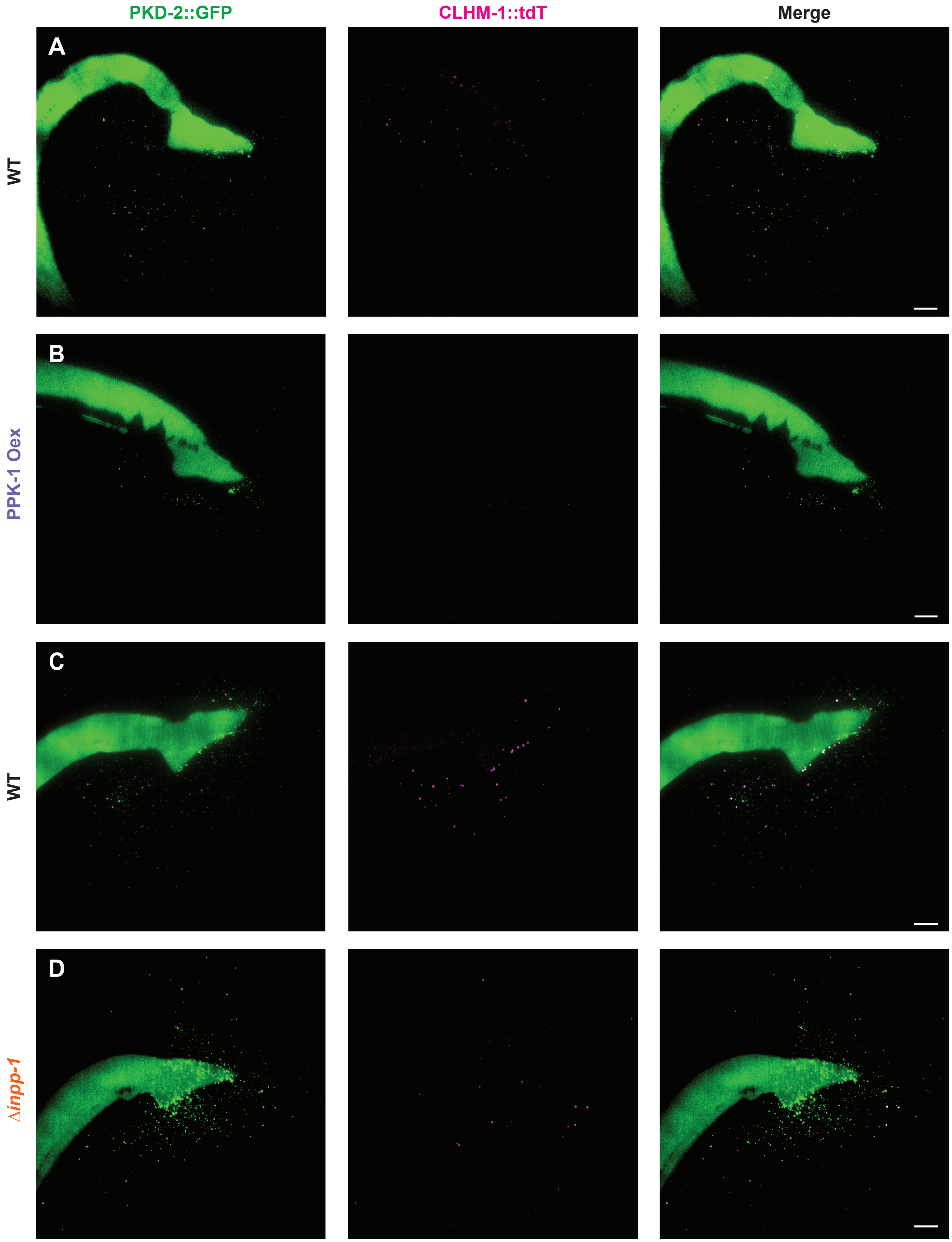
PKD-2::GFP
CLHM-1::tdT
Merge
A
WT
B
PPK-1 Oex
C
WT
D
Δinpp-1

Fig. S4. Altered abundance of PI(4,5)P_2 regulatory enzymes impacts EV shedding (A-D) Representative images of PKD-2::GFP (*henSi20*) and CLHM-1::tdT (*henSi3*) labeled-EVs released from (A,C) control, (B) PPK-1 Oex, and (D) *inpp-1* mutant male tails; scale, 10 µm. Images in A,B correspond to Fig. 3B,C; images in C,D correspond to Fig 5B,C.

Journal of Cell Science • Supplementary information

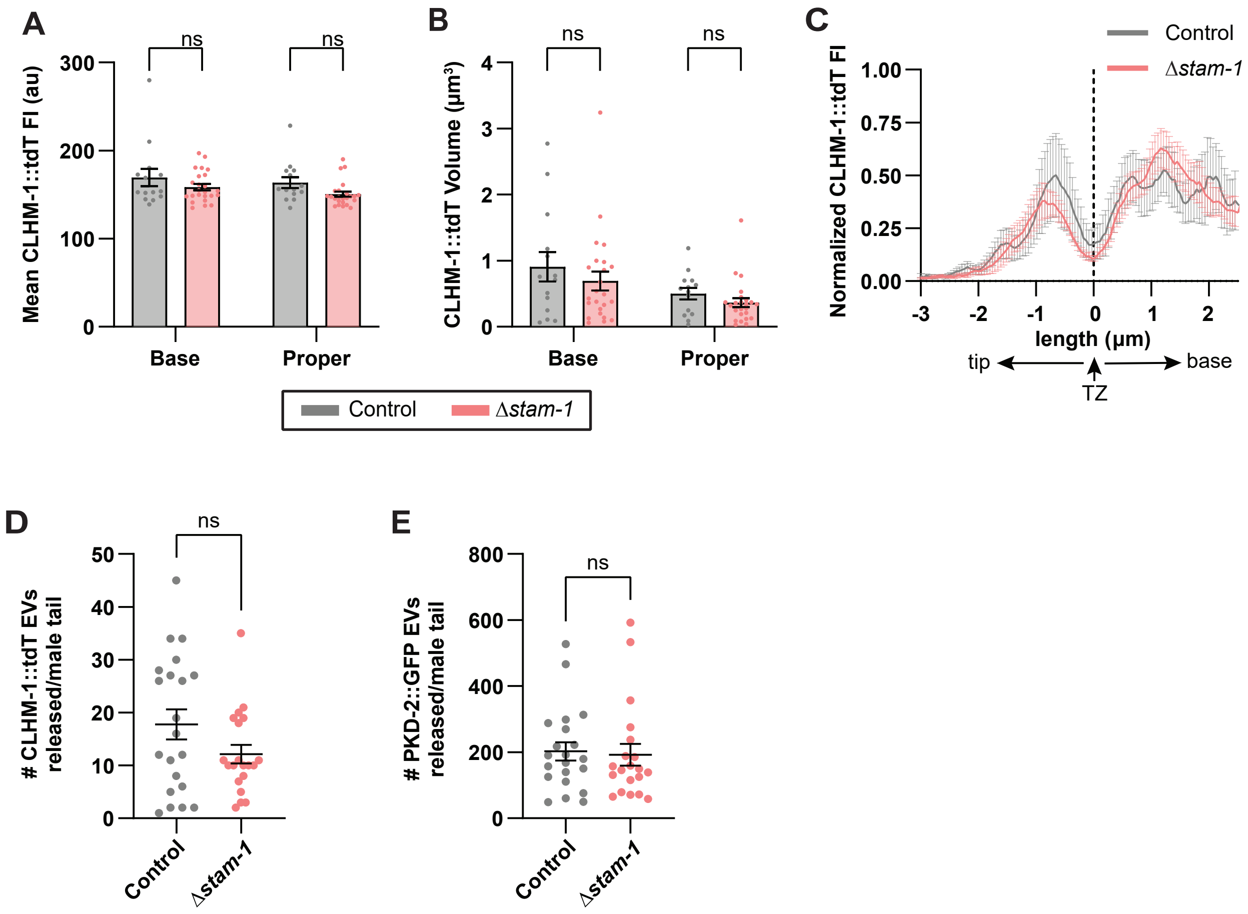

Fig. S5. Loss of STAM-1 does not affect CLHM-1 ciliary abundance, localization, or EV release. (A) Mean fluorescence intensity and (B) volume of CLHM-1::tdT in the ciliary base in control (grey) and *stam-1(ok406)* mutants (red); n ≥ 14. (C) Normalized CLHM- 1::tdT fluorescence intensity shows that loss of *stam-1* does not impact CLHM-1 distribution between the cilium proper and base. Dotted line indicates the TZ; n ≥ 14. (D) Release of CLHM- 1::tdT EVs and (E) PKD-2::GFP EVs from the *stam-1* mutant is not significantly different from the control; n ≥ 20. Data are represented as mean +/- SEM; Mann-Whitney test, ns = not significant.

Journal of Cell Science • Supplementary information

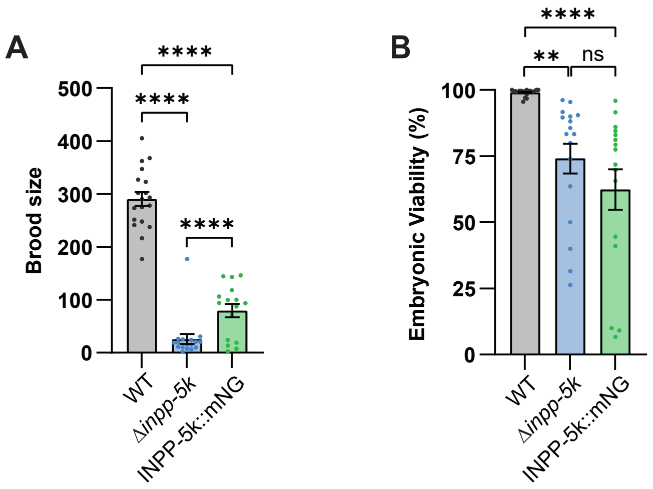

Fig. S6. The INPP-5K::mNG endogenous reporter retains partial function. (A) Brood size and (B) embryonic viability for wild-type control (grey), *inpp-5k(my15)* mutants (blue), and the INPP-5K::mNG(*syb9399*) endogenous reporter (green). Animals expressing INPP-5K::mNG have a significantly greater brood size compared to the *inpp-5k(my15)* mutant, but reduced brood size and embryonic viability compared to the wild type control, suggesting that insertion of the mNG C-terminal tag results in partial INPP-5K function. Data are presented as mean +/- SEM, one-way ANOVA, ** $p < 0.01$, **** $p < 0.0001$.

Journal of Cell Science • Supplementary information

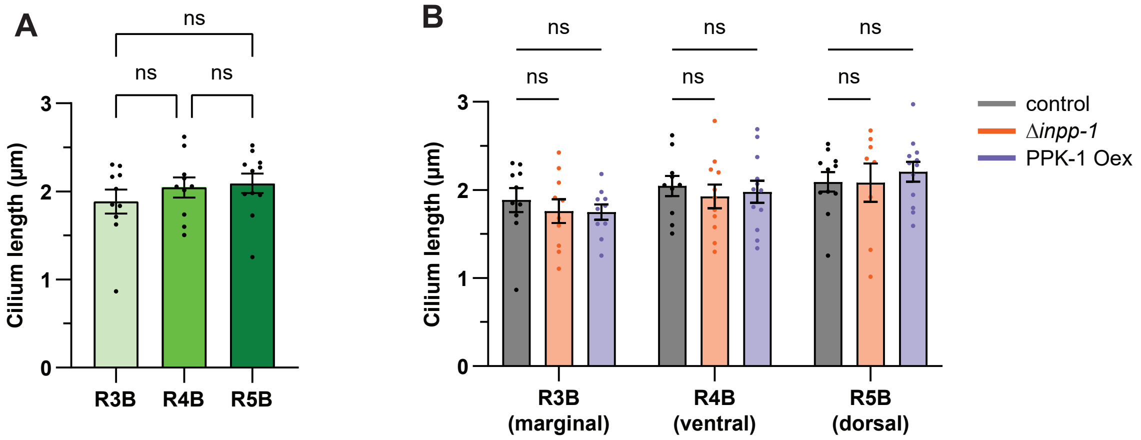

Fig. S7. Altered PI(4,5)P_2 does not impact cilium length (A) Length measurements of RnB3 (marginal opening), RnB4 (ventral opening), and RnB5 (dorsal opening) cilia in *him-5* control males expressing KLP-6::GFP with MKS-2::mSc; the cilia of these RnB neurons do not differ in length, n ≥ 8. (B) No significant difference in RnB3, RnB4, and RnB5 cilia lengths is observed between control (grey), n ≥ 10, *inpp-1* mutant (orange), n ≥ 8, and PPK-1 Oex (purple) animals, n ≥ 10. Data are represented as mean +/- SEM.

Table S1. Loss of *inpp-1* does not affect male mating behavior. Six parameters of male mating behavior were assessed for control and *inpp-1* mutant animals; both strains contain *him-5(e1490)*, which results in male offspring for analysis.

Indexes of mating behaviors	WT (n=10)	*inpp-1*(n=13)
Ventral contact duration, min	8.37 (0.78)	9.40 (0.99)
Vulva stop duration, min	2.12 (0.66)	2.28 (0.52)
Successful turns	14.90 (1.97)	23.00 (5.16)
Failed turns	2.40 (0.62)	2.69 (0.62)
Successful turns / min ventral contact	1.88 (0.30)	2.49 (0.34)
Failed turns / min ventral contact	0.29 (0.07)	0.28 (0.06)

Data are presented as mean ± SEM; all data are ns

Table S2. *C. elegans* strains used in this work

Strain	Alleles	Description
N2		*C. elegans* wild isolate
DR466	*him-5(e1490)* V	high incidence of males (~33%) due to increased frequency of X chromosome nondisjunction
UDE187	*inpp-1(gk3262)* IV; *him-5(e1490)* V	*inpp-1* deletion mutation removes two exons, resulting in a frameshift; presumed null mutation
PT1443	*inpp-5k(my15)* III; *him-5(e1490)* V	transition mutation which converts Trp301 in the 5th exon of *inpp-5k* into a stop codon.
UDE124	*stam-1(ok406)* I; *him-5(e1490)* V	*stam-1* deletion mutation; presumed null
PHX8819	*ppk-1(syb8819 [mNG::ppk-1])* I	CRISPR-generated mNeonGreen::PPK-1 translational fusion
PHX3371	*inpp-1(syb3371 [inpp-1::mNG])* IV	CRISPR-generated INPP-1::mNeonGreen translational fusion
PHX9399	*inpp-5k(syb9399)* III	CRISPR-generated INPP-5K::mNeonGreen translational fusion
PHX7299	*mks-2(syb7299 [mks-2::mSc])* II	CRISPR-generated MKS-2::mScarlet translational fusion
OEB912	*mks-2(oq101[mks-2::mNG])* II	CRISPR-generated MKS-2::mNeonGreen translational fusion
UDE381	*henIs1 [klp-6 promoter::mNG::PLCδ1-PH]* V	PI(4,5)P_2 reporter expressed in the EVNs
UF65	*gqIs25 [rab-3 promoter::ppk-1]*; *muIs32 [mec-7 promoter::GFP]*	Original strain with neuronal PPK-1 overexpression transgene
UDE238	*gqIs25 [rab-3 promoter::ppk-1]* I; *henSi21 [pkd-2 promoter::pkd-2::GFP::let858 3' UTR]* V; *him-5 (e1490)* V	PPK-1 overexpression transgene; strain also contains PKD-2::GFP single copy insertion transgene and *him-5* mutation
PT2332	*myIs10 [klp-6 promoter::klp-6::gfp]*	KLP-6::GFP translational fusion expressed in the EVNs
UDE50	*henSi3 [clhm-1 promoter::clhm-1::tdTomato ::let858 3'UTR]* III	CLHM-1::tdTomato single copy insertion
OG599	*drSi33 [clhm-1 promoter::clhm-1::gfp]* IV	CLHM-1::GFP single copy insertion
UDE103	*henSi20 [pkd-2 promoter::pkd-2::gfp:: let-858 3' UTR]* IV	PKD-2::GFP single copy insertion
UDE104	*henSi21[pkd-2 promoter::pkd-2::gfp:: let-858 3' UTR]* V	PKD-2::GFP single copy insertion
UDE372	*ppk-1(syb8819)* I; *mks-2(syb7299)* II; *him-5(e1490)* V	mNG::PPK-1 with MKS-2::mScarlet TZ marker and *him-5* mutation
UDE281	*mks-2(syb7299)* II; *inpp-1(syb3371)* IV; *him-5(e1490)* V	INPP-1::mNG with MKS-2::mScarlet TZ marker and *him-5* mutation
UDE414	*mks-2(syb7299)* II; *inpp-5k(syb9399)* III; *him-5(e1490)* V	INPP-5K::mNG with MKS-2::mScarlet TZ marker and *him-5* mutation

Journal of Cell Science • Supplementary information

UDE379	*mks-2(syb7299)* II; *henIs1* V	PI(4,5)P_2 reporter with MKS-2::mScarlet TZ marker
UDE382	*gqIs25* I; *mks-2(syb7299)* II; *henIs1* V	PPK-1 overexpression transgene with PI(4,5)P_2 reporter and MKS-2::mScarlet TZ marker
UDE383	*mks-2(syb7299)* II; *inpp-1(gk3262)* IV; *henIs1* V	*inpp-1* deletion mutation with PI(4,5)P_2 reporter and MKS-2::mScarlet TZ marker
UDE409	*mks-2(syb7299)* II; *inpp-5k(my15)* III; *henIs1* V	*inpp-5k* early stop mutation with PI(4,5)P_2 reporter and MKS-2::mScarlet TZ marker
UDE310	*henSi3* III; *henSi21* V; *him-5(e1490)* V	CLHM-1::tdT and PKD-2::GFP single copy insertion (SCI) transgenes with *him-5* mutation; used to visualize EV release
UDE237	*gqIs25* I; *henSi3* III; *henSi21* V; *him-5(e1490)* V	PPK-1 overexpression transgene with CLHM-1::tdT and PKD-2::GFP SCI transgenes and the *him-5* mutation; used to visualize EV release
UDE249	*mks-2(oq101)* II ; *henSi3* III; *him-5(e1490)* V	CLHM-1::tdT SCI transgene with MKS-2::mNG TZ marker and *him-5* mutation; used to visualize CLHM-1 ciliary localization
UDE245	*gqIs25* I; *mks-2(oq101)* II; *henSi3* III; *him-5(e1490)* V	PPK-1 overexpression transgene with CLHM-1::tdT SCI, MKS-2::mNG TZ marker, and *him-5* mutation; used to visualize CLHM-1 ciliary localization
UDE425	*ppk-1(syb8819)* I; *mks-2(oq101)* II; *henSi3* III; *him-5(e1490)* V	mNG::PPK-1 fusion, which causes loss of PPK-1 function, with CLHM-1::tdT SCI, MKS-2::mNG TZ marker, and *him-5* mutation; used to visualize CLHM-1 EV release and ciliary localization
UDE332	*mks-2(syb7299)* II; *henSi21* V; *him-5(e1490)* V	PKD-2::GFP SCI transgene with MKS-2::mSc TZ marker and *him-5* mutation; used to visualize PKD-2 ciliary localization
UDE334	*gqIs25* I; *mks-2(syb7299)* II; *henSi21* V; *him-5(e1490)* V	PPK-1 overexpression transgene with PKD-2::GFP SCI, MKS-2::mSc TZ marker, and *him-5* mutation; used to visualize PKD-2 ciliary localization
UDE288	*stam-1(ok406)* I; *henSi3* III; *henSi21* V; *him-5(e1490)* V	*stam-1* deletion mutation with CLHM-1::tdT and PKD-2::GFP SCI transgenes and the *him-5* mutation; used to visualize EV release and CLHM-1 ciliary localization
UDE275	*henSi3* III; *inpp-1(gk3262)* IV; *henSi21* V; *him-5(e1490)* V	*inpp-1* deletion mutation with CLHM-1::tdT and PKD-2::GFP SCI transgenes and the *him-5* mutation; used to visualize EV release
UDE340	*mks-2(syb7299)* II; *inpp-1(gk3262)* IV; *henSi21* V; *him-5(e1490)* V	*inpp-1* deletion mutation with PKD-2::GFP SCI, MKS-2::mSc TZ marker, and *him-5* mutation; used to visualize PKD-2 ciliary localization
UDE302	*mks-2(syb7299)* II; *drSi33* IV; *him-5(e1490)* V	CLHM-1::GFP SCI with MKS-2::mScarlet TZ marker and *him-5* mutation; used to visualize CLHM-1 EV release and ciliary localization
UDE412	*mks-2(syb7299)* II; *inpp-5k(my15)* III; *drSi33* IV; *him-5(e1490)* V	*inpp-5k* mutation with CLHM-1::GFP SCI, MKS-2::mScarlet TZ marker, and *him-5* mutation; used to visualize CLHM-1 EV release and ciliary localization

Journal of Cell Science • Supplementary information

UDE344	*mks-2(syb7299)* II; *henSi20* IV; *him-5(e1490)* V	PKD-2::GFP SCI with MKS-2::mScarlet TZ marker and *him-5* mutation; used to visualize PKD-2 EV release and ciliary localization
UDE411	*mks-2(syb7299)* II; *inpp-5k(my15)* III; *henSi20* IV; *him-5(e1490)* V	*inpp-5k* mutation with PKD-2::GFP SCI, MKS-2::mScarlet TZ marker, and *him-5* mutation; used to visualize PKD-2 EV release and ciliary localization
UDE369	*mks-2(syb7299)* II; *him-5(e1490)* V; *myIs10*	KLP-6::GFP with MKS-2::mScarlet TZ marker and *him-5* mutation; used to measure cilium length
UDE360	*mks-2(syb7299)* II; *inpp-1(gk3262)* IV; *him-5(e1490)* V; *myIs10*	*inpp-1* deletion mutation with KLP-6::GFP, MKS-2::mScarlet TZ marker and *him-5* mutation; used to measure cilium length
UDE333	*gqIs25* I; *mks-2(syb7299)* II; *him-5(e1490)* V; *myIs10*	PPK-1 overexpression transgene with KLP-6::GFP, MKS-2::mScarlet TZ marker and *him-5* mutation; used to measure cilium length

Journal of Cell Science • Supplementary information
